# Characterization of Metabolomic Response of *Candida* spp. to Heavy Metal Exposure

**DOI:** 10.3390/microorganisms13102394

**Published:** 2025-10-19

**Authors:** Perla Nayeli Reyes-Sánchez, Jesús Alfonso Chairez-Ávila, Karol Karla García-Aguirre, Verónica Esparza-Cordero, María Fernanda Romo-García, Juan C. Medina-Llamas, Juan Ernesto López-Ramos

**Affiliations:** 1 Unidad Profesional Interdisciplinaria de Ingeniería Campus Zacatecas, Instituto Politécnico Nacional, Zacatecas 98160, Zacatecas, Mexico; 2 Laboratorio de Inmunotoxicología, Universidad Autónoma de Zacatecas, Zacatecas 98160, Zacatecas, Mexico; 3 Centro de Estudios Científicos y Tecnológicos 18, Instituto Politécnico Nacional, Zacatecas 98160, Zacatecas, Mexico

**Keywords:** *Candida* spp., heavy metals, secretome characterization, GC-MS

## Abstract

As a result of anthropogenic activities, contaminants such as heavy metals have been introduced into the environment. Microorganisms, including *Candida* spp., have emerged as a viable alternative for their removal; however, the mechanism responsible for the removal process has not been fully characterized. This study aims to identify the secretome of *Candida* strains (*C. albicans, C. glabrata, C. parapsilosis* and *C. tropicalis)* contributing to their ability to withstand heavy metals. For this purpose, *Candida* spp. cultures were incubated at 28 °C under agitation for 72 h and exposed to different concentrations of Cd^2+^, Cu^2+^ and Zn^2+^. The cultures were then analyzed using GC-MS. In *Candida* spp. cultures exposed to heavy metals, 117 different compounds were identified compared with the control cultures. Among all *Candida* spp., 15 metabolites showed a fold change greater than two compared to the control conditions. These included hydrocarbons (3), fatty acids (5), aromatic compounds (5), a nonaromatic compound (1) and an organosiloxane (1), which were identified in the presence of heavy metals (Cd, Cu and Zn).

## 1. Introduction

Heavy metals are naturally occurring elements, most of which share certain characteristics, for example, an atomic number (Z) greater than 20; an elemental density greater than 5 g/cm^3^; and toxicity to humans, animals and plants [1]. Regarding toxicity, one of the main mechanisms of metabolic disruption occurs through the impairment of mitochondrial respiratory complexes. In this process, divalent metal cations such as Cd^2+^ and Cu^2+^ can accumulate within mitochondria via the Ca^2+^ uniporter channel, substituting protein metallic cofactors, increasing the production of reactive oxygen species (ROS), or oxidizing catalytic residues of SH groups. This leads to an increased permeability of cellular membranes [2]; as a fingerprint of this metabolic pathway, lipid hydroperoxides, including lipid alkoxyl radicals, aldehydes (e.g., malondialdehyde) and lipid epoxides, can be produced [3].

However, the metabolites produced during heavy metal removal have not been fully described. One of the principal heavy metal removal mechanisms is surface sorption, which includes physical and chemical interactions [4]. This process is highly dependent on membrane characteristics, and it has recently been shown that new strains with modified cell membranes exhibit improved xenobiotic degradation and heavy metal reduction [5].

Microorganisms have evolved different detoxifying mechanisms, mainly enabling them to mobilize metal ions through biosorption and bioaccumulation by excreting acids and fatty acids that can interact with metal ions and produce insoluble complexes or modify membranes [6]. For example, metallophiles and metal-resistant microorganisms can survive at high concentrations of metals such as cadmium, silver, mercury and lead. The most studied metallophile microorganisms are bacteria, especially Gram-negative [7]; in contrast, information regarding metal resistance on fungal species is limited. In a general context, some studies have shown that ascomycetous fungi are more tolerant to heavy metals than basidiomycetous fungi [8], and some yeast species can survive under extreme conditions in contaminated environments [9].

As mentioned previously, several yeasts have demonstrated particular tolerance to various substances, such as the manganese-resistant strain of *Saccharomyces cerevisiae* [10] and *Pichia guilliermondii*, which is resistant to Mn, Zn and Co [11]. In particular, *Candida* spp. exhibit metal resistance, for example, *Candida parapsilosis* to Ni, Zn, Cu, Cr, Pb and Hg [12]; *Candida krusei* tolerates high concentrations of Zn (up to 20 mM) [8] and is also capable of removing Cu [13]. Nevertheless, unlike these examples, limited information is available regarding the metabolites secreted or produced by *Candida* spp. under heavy metal stress conditions. In this study, we evaluated the metabolite profiles of *Candida albicans*, *Candida glabrata*, *Candida parapsilosis* and *Candida tropicalis* when exposed to Cd, Cu and Zn.

Cadmium is a toxic element that is widely distributed in the environment, primarily due to anthropogenic activities [14]. It can accumulate in human tissues following environmental exposure, particularly through contaminated water and soil [15]. Notably, elevated Cd concentrations have been reported in water sources in regions of Korea and China, where irrigation of rice fields represents a potential route of human exposure [16,17]. Copper, in contrast, is a naturally occurring element and an essential nutrient for many organisms; however, at elevated concentrations, it can become potentially toxic. It has been reported that certain organisms, such as *Candida albicans*, possess mechanisms that enable them to withstand copper-induced oxidative stress through the expression of cytosolic superoxide dismutase (Sod1) [18]. On the other hand, zinc is an essential trace element widely present in the environment and serves as a key cofactor for numerous eukaryotic proteins (approximately nine percent of them). It has also been demonstrated that certain microorganisms, such as *Candida albicans*, secrete a zinc-scavenging protein known as the zincophore Pra1, which sequesters zinc from host cells and subsequently re-associates with the fungus through the co-expressed zinc transporter Zrt1 [19].

Some mechanisms associated with metal tolerance in *Candida* spp. involve the generation of a broad spectrum of reactive oxygen species during the oxidative burst, together with antioxidant defense systems such as catalases, superoxide dismutases, thioredoxins, glutathione-dependent peroxidases and reductases [20].

*Candida* spp. strains are resistant to heavy metal toxicity due to their intrinsic resistance to ROS, expressing CaSod4 and CaSod5 superoxide dismutase enzymes involved in metal survival, which have an antioxidant role, as well as stress-related transcription factors encoding putative orthologues of Yap1 and Skn7 [21]. The genetic signature of *Candida* tolerance to metal-induced stress is well documented; however, there is limited information about metabolites involved in this response. In this context, López Ramos [22] reported changes on secretome of *Candida* spp. cultured in different growth media, identifying compounds such as fatty acids (nonanoic acid and eicosane) and aromatic alcohols (phenylethanol, tyrosol and tryptophol).

In this study, we characterized the metabolite profile secreted by *Candida* spp. when exposed to cadmium (Cd), copper (Cu), and zinc (Zn), in order to evaluate their response to heavy metal-induced stress. We analyzed the composition and variation in secreted metabolites to understand the adaptative process that enable *Candida* spp. to tolerate and respond to toxic metal environments.

## 2. Materials and Methods

### 2.1. Cell Growth and Culture Conditions

The strains were obtained from the American Type Culture Collection (ATCC), as detailed in Table 1. Initially, the LD50 was determined in YPD medium (2% peptone MCD lab, San Jacinto Amilpas, Mexico), 1% yeast extract (MCD lab, San Jacinto Amilpas, Mexico) and 2% dextrose (CTR laboratories, Monterrey, Mexico)) at 28 °C and 120 rpm for 12 h, using various concentrations of heavy metals: Cd^2+^ (1 mM to 15 mM, J.T. Baker, Phillipsburg, PA, USA), Cu^2+^ (1 mM to 21 mM, J.T. Baker, Phillipsburg, PA, USA) and Zn^2+^ (1 mM to 1200 mM, J.T. Baker, Phillipsburg, PA, USA). After incubation, serial 1:10 dilutions were performed up to 1:10,000 in 0.85% saline solution. Cell viability was assessed using the modified drop plate method [23], by inoculating 10 µL of each dilution onto YPD plates in triplicate and incubating at 28 °C for 48 h. Cell viability was expressed as colony-forming units (CFU) per milliliter and the LD_50_ was determined for each condition [24].

Subsequently, the *Candida* spp. strains were cultured in YPD medium (2% peptone, 1% yeast extract and 2% dextrose) at 28 °C and 120 rpm for 72 h. Afterward, they were exposed to the LD50 concentrations of Cd^2+^, Cu^2+^ and Zn^2+^. The culture of each *Candida* sp. was grown under the same conditions described above, but without heavy metals, to serve as a control.

#### Sample Preparation for GC-MS Analysis

Sample preparation for GC-MS analysis was performed as described by López-Ramos et al. (2021) [22]. Samples of *C. albicans*, *C. glabrata*, *C. parapsilosis, C. tropicalis*, and the control (culture medium without cells) were extracted in triplicate, with each being considered an independent sample. Briefly, 10 mL of cell-free supernatants were extracted with 10 mL of chloroform (Fisher Scientific, Santa Clara, CA, USA). The chloroform layer was dehydrated with anhydrous sodium sulfate (Fisher Scientific), passed through a 0.2 µm syringe filter and evaporated in a fume hood. The material from each extract was dissolved in 500 µL of chloroform. Then, for each sample, 100 µL of the chloroform extract was derivatized with 50 µL of N-trimethylsilyl-N-methyl trifluoroacetamide and trimethylchlorosilane (MSTFA +1% TMS, Sigma-Aldrich, St. Louis, MO, USA) prior to GC-MS analysis.

### 2.2. Characterization

GC-MS analysis was performed at the Instituto Potosino de Investigación Científica y Tecnológica A.C. (IPICYT) in San Luis Potosí using a 7890B gas chromatograph coupled to a 5977A mass selective detector, both manufactured by Agilent Technologies (Santa Clara, CA, USA). An HP-5ms UI capillary column (30 m × 0.25 mm ID × 0.25 µm film thickness) was employed. Helium (99.9995% purity) was used as the carrier gas at a flow rate of 0.78371 mL/min. The oven temperature was initially set to 70 °C for 10 min, then increased at a rate of 10 °C/min to 200 °C, where it was held for an additional 10 min. The injection volume was 0.2 µL at 280 °C in splitless mode. The mass spectrometer operated in electron impact mode with an average energy of 70 eV. The ion source temperature was 230 °C, and the interface temperature was 200 °C. Mass spectra were acquired in full scan mode over an *m*/*z* range from 20 to 500. Data acquisition and analysis were performed using MSD Enhanced ChemStation software (Agilent Technologies, Santa Clara, CA, USA, https://www.agilent.com.cn/en-us/support/software-informatics/multiinstrumentsoftwarerev, accessed on 1 January 2024). Compound identification was achieved by comparing the obtained spectra with the W10N11 database.

### 2.3. GC-MS Analysis Results

Chromatograms representing the compounds present in the liquid medium were obtained. Each chromatogram included a similarity score (Qual), indicating the degree of match to potential compounds. To ensure data quality, only compounds with a “Qual” score above 60% were considered. The total area for each compound was calculated to determine its relative abundance in the medium.

Once all compounds were identified, a pre-selection strategy was employed to focus on the most significant ones. This was achieved by subtracting the basal total area (from non-exposed cultures) from the exposed cultures, and then calculating the fold change to determine the increase or decrease in compound levels compared to the control groups.

### 2.4. Statistical Analysis

A Spearman correlation analysis was conducted to evaluate the association between the metabolites produced by *Candida* spp. (*Candida albicans*, *Candida glabrata*, *Candida parapsilosis* and *Candida tropicalis*) after exposure to heavy metals (Cu, Cd and Zn). The analysis was performed in RStudio 2025.09.1+40 using the function cor. (method = “spearman”), and the command rcorr(as.matrix()) was applied to obtain the correlation matrix. Subsequently, the heatmap function was used to generate graphical representation. Only compounds exhibiting a ratio greater than 2 or less than –2 were included in the analysis to ensure meaningful differential expression. Heatmaps for each *Candida* strain and metal condition were subsequently generated using GraphPad Prism v8.0 to visualize the distribution and relative abundance of these metabolites. Venn diagrams were constructed using the jvenn web tool BMC Bioinformatics 2014 (https://jvenn.toulouse.inrae.fr/app/example.html, accessed on 1 January 2025) to visualize the number of compounds shared by the different strains when exposed to the metals of interest. Chemical structures of the identified compounds were drawn with Chem Draw Professional 17.0 software.

## 3. Results

LD50 concentrations were determined to incubate *Candida* spp. strains under conditions that induced significant stress in these yeasts, while maintaining cell mortality at a level that did not completely eliminate the viable population. This allowed for subsequent analysis of the secretome under relevant stress conditions. Table 2 shows the LD_50_ values for each *Candida* strain and metal. At these concentrations, stress and cell death occurred but did not exceed 50% of the population.

### Metabolic Profile

**Figure 1:**
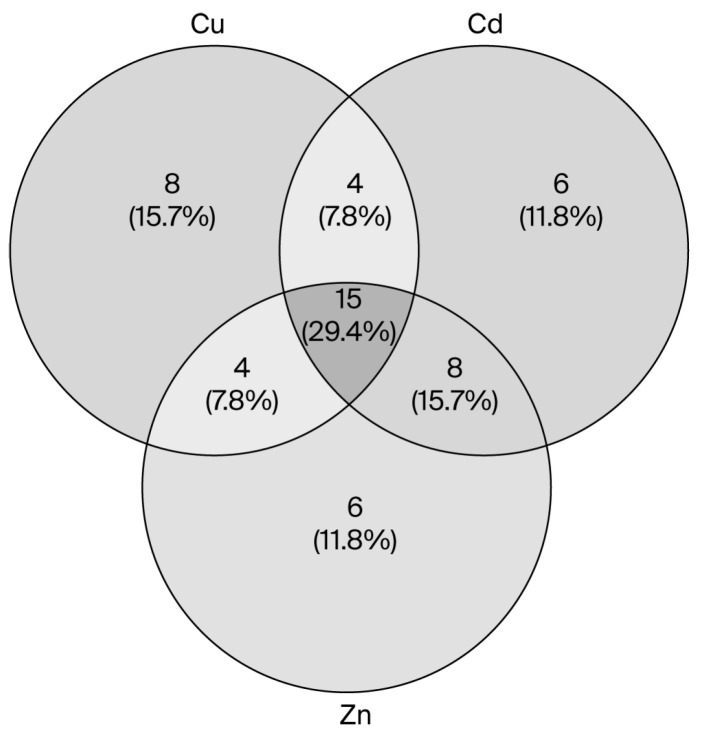
Venn diagram illustrating compounds modified by exposure to different heavy metals in *Candida* strains. The diagram was generated using the Venny tool 2.1, at: https://bioinfogp.cnb.csic.es/tools/venny/index.html (accessed on 1 April 2025).

A total of 84 metabolites were identified, excluding those expressed in the metabolomic profiles of control cultures. Of these, 15 compounds were exclusively overproduced or decreased in all *Candida* strains exposed to heavy metals compared to control strains, representing 29.4% of the total compounds included in the analysis (Figure 1). Copper and zinc are heavy metals that are well tolerated by yeast and other microorganisms. The metabolites identified in strains exposed to each metal are shown in Table 3. In the presence of copper, six metabolites were produced by the *Candida* strains, which were not observed in cultures exposed to Cd. For zinc, the metabolites were 4-(3-Pyridyl)thieno [2,3-d]pyridazin-7(6H)-one, 9-Azatetracyclo[10.2.1.0(2,11).0(3,8)]pentadeca-3,5,7-triene-7-carboxylic acid, 10-(2,3-difluorophenyl), E-15-Heptadecenal, Hexacosane and Hexadecane-1-iodo. Since zinc is not classified as a toxic metal for *Candida* spp., these metabolites could be considered basal, with only four metabolites detected as being shared between Cd and Cu (toxic metals), namely, (1S,3S)-6,8-dimethoxy-1,3-dimethyl-3,4-dihydro-1H-2-benzopyran-5-ol, benzoic acid, heneicosane and N-(1,1,Dimethylethyl)-2-(2-thienyl)-4-quinolinamine, suggesting that these metabolites could be related to processes of heavy metal resorption or response to metal stress.

**Figure 2:**
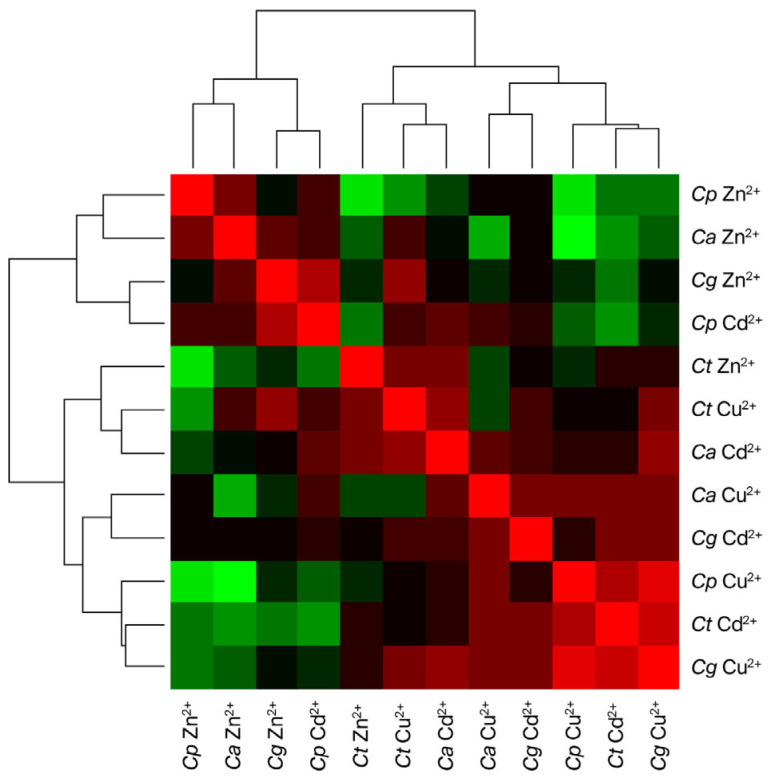
Cluster heatmap showing the correlation between *Candida* spp. and heavy metals. The color gradient transitions from green, representing lower Spearman correlation values, to red, indicating higher correlation values, with black denoting intermediate levels of correlation. The cluster analysis groups correlated the variables based on the minimal differences in correlation patterns.. The heatmap was generated using the heatmap function in RStudio (version 2025.09.1+40).

The correlations among the metabolites expressed by *Candida* spp. were examined and are presented in Figure 2. When evaluating the compounds secreted by *Candida parapsilosis* and *Candida glabrata* in the presence of Zn, these species exhibited metabolic profiles that were clearly distinct from that of *Candida tropicalis*. In contrast, under Cd- and Cu-induced stress conditions, the metabolite profiles of *Candida* spp. did not display a consistent correlation, suggesting a species-specific response to Zn exposure that is not observed for Cd or Cu.

**Figure 3:**
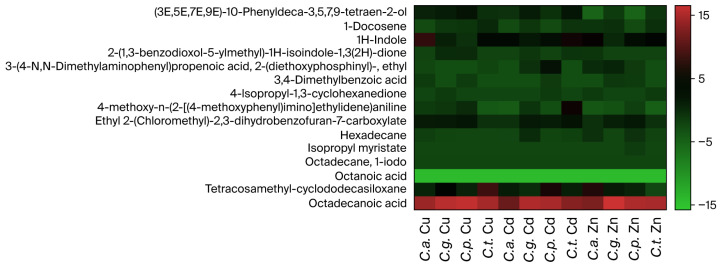
Heatmap depicting the fold change values (ranging from −15 to 15) for metabolite production higher than 2 times compared to non-exposed cultures. There are 15 metabolites shared among *Candida* spp. strains exposed to Zn^2+^, Cd^2+^ and Cu^2+^. The red color indicates a higher fold change in expression, whereas colors closer to green represent lower fold change. The strains include *Candida albicans* (*C.a.*), *Candida glabrata* (*C.g*.), *Candida tropicalis* (*C.t.*) and *Candida parapsilosis* (*C.p.*).

**Figure 4:**
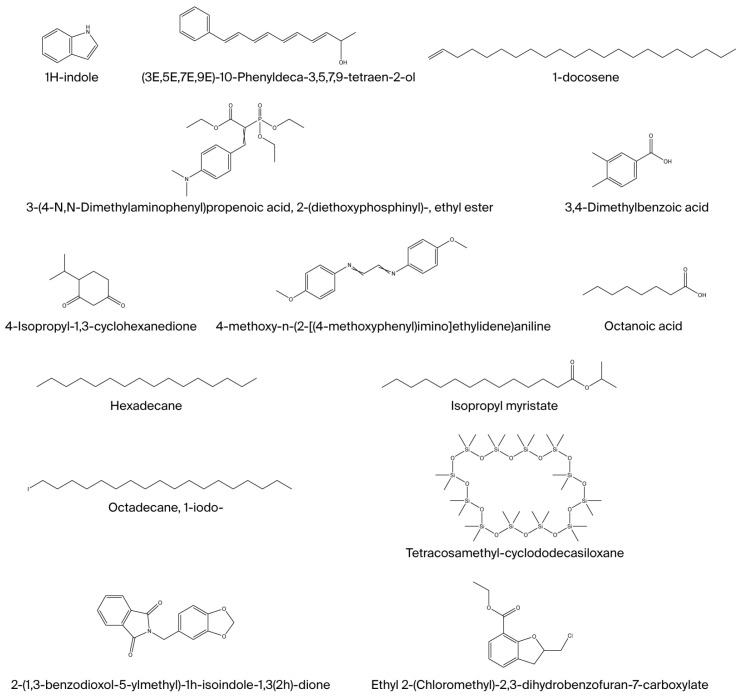
Chemical structures of metabolites detected in *Candida albicans, C. tropicalis, C. parapsilosis* and *C. glabrata* following exposure to heavy metals, drawn using ChemDraw Professional 17.0.

The heatmap illustrates the relative changes in compound production in *Candida* cultures exposed to heavy metals compared to their respective control. The compounds that showed increased production were octadecanoic acid and 1H-indole (Figure 3). Conversely, octanoic acid, isopropyl myristate and octadecane-1-iodo exhibited decreased production in *Candida* cultures exposed to heavy metals. The 15 compounds identified in all *Candida* strains (*Candida glabrata*, *Candida parapsilosis*, *Candida albicans* and *Candida tropicalis*) are described in Figure 4. In addition, Table 4 was created to present the classification of the identified compounds according to their chemical composition and the corresponding quality score values.

Based to results, Zn appears to specifically repress the production of (3E,5E,7E,9E)-10-phenyldeca-3,5,7,9-tetraen-2-ol in both *Candida albicans* and *Candida parapsilosis*. Furthermore, Zn, Cu and Cd all appear to reduce the production of 4-Methoxy-N-(2-[(4-methoxyphenyl)imino]ethylidene).

**Figure 5:**
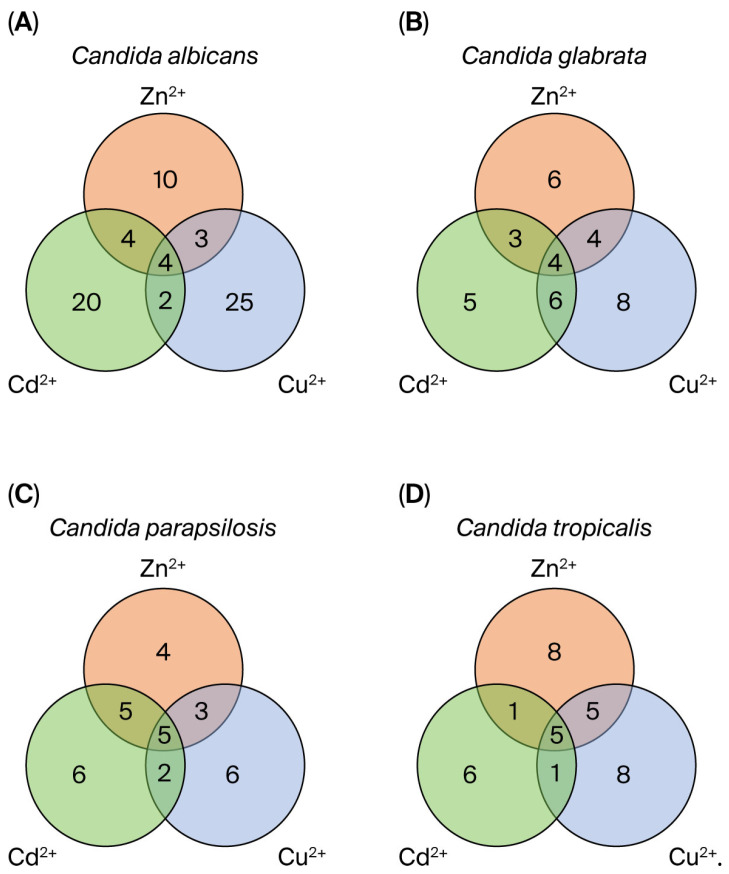
Venn diagrams illustrating metabolites identified in each *Candida* strain following exposure to heavy metals. Metabolites expressed by *Candida albicans* (**A**), *Candida glabrata* (**B**), *Candida parapsilosis* (**C**) and *Candida tropicalis* (**D**) when heavy metals (Zn^2+^, Cu^2+^ and Cd^2+^) were added to the culture medium. The diagrams were generated using the Venny tool, at https://jvenn.toulouse.inrae.fr/app/example.html (accessed on 1 June 2025).

To assess the number of metabolites produced by the *Candida* strains in the presence of heavy metals (Cu^2+^, Cd^2+^ and Zn^2+^), Venn diagrams were generated to identify such compounds. Figure 5 illustrates the metabolites shared among the strains of *Candida albicans*, which include 1H-Indole, 3-(2-Hydroxyethyl)indole, octadecanoic acid and 4-Hydroxyphenylethanol; for *Candida glabrata*, they were 1H-Indole, Ethyl 2-(Chloromethyl)-2,3-dihydrobenzofuran-7-carboxylate, octadecane and octadecanoic acid; for *Candida tropicalis*, they were 1H-Indole, 3-(2-Hydroxyethyl)indole, 4-Hydroxyphenylethanol, octadecane and octadecanoic acid; for *Candida parapsilosis*, they were 3-(2-Hydroxyethyl)indole, 4-Hydroxyphenylethanol, ethyl 2-(Chloromethyl)-2,3-dihydrobenzofuran-7-carboxylate, octadecane and octadecanoic acid.

## 4. Discussion

Previous studies have reported that *Candida* spp. can be used for the bioremediation of pollutants [26] through several processes, some of which are receptor-dependent or associated with membrane modifications. For example, *Candida tropicalis* has demonstrated the ability to degrade diesel oil and azo dye Acid Brilliant Scarlet GR [27,28], while *Candida glabrata* has been used in the remediation of motor oil [29]. These capabilities are not exclusive to these compounds, as similar bioremediation potential has been reported for heavy metals. For instance, *Candida utilis* has been shown to remove Cu from mining industry waste [30], and applied to the removal of textile dyes [26]. Likewise, *Candida tropicalis* has been investigated as a microbial agent for the removal of Cd in both the mining and fertilizer industries [30], while *Candida albicans* has demonstrated the ability to remove As, Pb, Hg and Cr [26].

*Candida tropicalis* isolated from wastewater effluents has reportedly demonstrated the ability to remove 40% and 78% of Cd from contaminated water after 6 and 12 days, respectively. This process is associated with an increase in the production of glutathione (GSH), a metabolite presumably relevant to *Candida tropicalis* tolerance to Cd exposure. These results indicate that *C. tropicalis* can serve as an effective agent for the removal of Cd from wastewater effluents [31] and further suggest that heavy metal exposure alters the metabolite production as part of the cellular response of *Candida* spp.

In the present study, we identified octadecanoic acid (stearic acid) as a compound produced by *Candida albicans*, *Candida parapsilosis, Candida tropicalis* and *Candida glabrata* that may contribute to cell ability to survive the stress generated by the presence of Cu, Cd and Zn, possibly through membrane modifications. It is known that octadecanoic acid is a signal-inducing molecule in response to biotic stress in the case of plants [32]. Although the biosynthesis of saturated and monounsaturated long-chain fatty acids (C16-C18) has been reported [33], information about the role of this metabolite in yeast remains limited. Previous studies using GC-MS analysis have shown high levels of fatty acid in biosurfactants, with 58% of identified metabolites corresponding to lipids present in the sample, specifically capric acid (C8:0), palmitoleic acid (C16:0), stearic acid (C18:0), oleic acid (C18:1) and linoleic acid (C18:2) [34].

Octanoic acid had been associated with certain stress conditions; for example, in *Saccharomyces cerevisiae,* overexpression of OLE1 has been correlated with cadmium stress. This gene encodes delta-9 desaturase, which produces saturated fatty acids from endogenous monounsaturated fatty acids, such as oleic acid (octadecanoic acid), one of the main components of cell membranes. It has also been observed that OLE1 can inhibit cadmium-induced lipid peroxidation by increasing the production of unsaturated fatty acids, which may exert antioxidant functions [35]. Furthermore, in *Saccharomyces cerevisiae*, the fatty acid biosynthesis in the cytosol is catalyzed by the large, multidomain fatty acid synthase (FAS) complex, which naturally generates long-chain fatty acids (LCFA, C14-C18) as building blocks of membranes or storage lipids; the biosynthetic process is initiated by cytosolic acetyl-CoA (AcCoA), and the elongation of FAs continues via AcCoA-derived malonyl-CoA until reaching the final length [36]. Similarly, the presence of heavy metals in *Candida tropicalis, Candida albicans, Candida parapsilosis* and *Candida glabrata* has been associated with the induction of fatty acid production as a cellular adaptation mechanism. Moreover, the antibiofilm activity of medium-chain fatty acids against *Candida albicans* has been described [37], suggesting a possible adaptive response by *Candida* spp. to environmental stress such as heavy metal exposure.

On the other hand, a compound detected at lower concentrations compared to the control was octanoic acid, a medium-chain fatty acid produced in yeast via the fatty acid synthesis cycle through the action of cytosolic fatty acid synthase, encoded by the *FAS1* and *FAS2* genes [38]. This metabolite is considered toxic to yeast; for example, the carboxylic acids hexanoic, octanoic and decanoic acid inhibit the growth of *Saccharomyces cerevisiae* at concentrations around 1 mM due to membrane disruption [39].

In addition, octanoic acid inhibits growth, decreases biomass production and increases ATP levels. The metabolic alterations induced by octanoic acid are associated with changes in intracellular pH (pHi). Exposure to inhibitory concentrations (up to 44.5 mgL^−1^) result in the decreased synthesis of succinic and acetic acids in octanoic acid-stressed cells, as well as an increased cytoplasmic buffering capacity due to a reduced internal volume [40]; in the case of *Candida* spp., fatty acid production has also been shown to be affected by the presence of n-alkanes [41]. Decrease in octanoic acid production appears to be an adaptive response to metal-induced stress, likely due to the downregulation of membrane-disrupting metabolites such as octanoic acid. Conversely, metabolites associated with adaptive mechanisms that counteract oxidative damage and membrane lipid disruption, such as octadecanoic acid, are overproduced. Octadecanoic acid probably modifies the transport system, including transport via lipid rafts, which plays an important role in membrane transport and in maintaining the proper fluidity of the membrane. Lipid rafts are domains rich in sterols and sphingolipids that serve several functions, including ensuring correct intracellular trafficking of lipids and proteins, sorting and vesicle formation, as well as signaling and vesicle uptake [42].

In addition, most of the compounds produced by *Candida* spp. and identified in this study are classified as antifungal agents. For example, gas chromatography/mass spectrometry analysis of passion fruit ethanol extracts showed the presence of several compounds, including tetracosamethylcyclododecasiloxane; dodecanoic acid, 10-methyl-, methyl ester cyclosiloxane, hexadecamethyl; 3-isopropoxy-1,1,1,7,7,7-hexamethyl-3,5,5-tris (trimethylsiloxy)tetrasil; 9-hexadecenoic acid, 9-octadecenyl ester. Compounds such as tetracosamethylcyclododecasiloxane, cyclosiloxane and hexadecamethyl have demonstrated antibacterial, antifungal and antioxidant properties [43]. In addition, several microorganisms have been shown to convert L-phenylalanine and/or (E)-cinnamate into both benzoic acids and benzaldehydes [44]. Benzaldehyde, in particular, has been identified as one of the volatile components in the fruiting body of *Tricholoma matsutake* [45].

## 5. Conclusions

The results reveal a profile of metabolites detected in *Candida* spp. in the presence of heavy metals; a few metabolites are also produced by other yeast species in response to oxidative stress induced by heavy metals (Cd, Cu and Zn). The main compounds identified were classified into categories, including hydrocarbons, fatty acids, aromatic compounds, non-aromatic compounds and organosiloxanes. Nevertheless, further research is required to determine whether these metabolites are produced as part of an adaptive process or function as a defense mechanism against the presence of heavy metals, as well as to specifically evaluate this role in *Candida* spp.

## Figures and Tables

**Table 1:** Strains of *Candida* spp. used in this study.

Genotype	Strain	Source of Reference
*Candida glabrata* strain BG2: *ura3*Δ:Tn*903* Neo^R^ Ura^−^	BG14	[25]
*C. parapsilosis*	ATCC-22019	De Las Peñas’s Lab. collection
*C. tropicalis*	ATCC-750	De Las Peñas’s Lab. collection
*C. albicans* Sc5314	ATCC MYA2876	De Las Peñas’s Lab. collection

**Table 2:** LD_50_ values for the strain each *Candida* sp. exposed to Zn^2+^, Cd^2+^ and Cu^2+^.

Metal	Strain	LD_50_ (mM)
Cd^2+^	*C. albicans*	0.03
	*C. glabrata*	2
	*C. parapsilosis*	1.5
	*C. tropicalis*	0.2
Cu^2+^	*C. albicans*	6
	*C. glabrata*	7
	*C. parapsilosis*	3.5
	*C. tropicalis*	5
Zn^2+^	*C. albicans*	400
	*C. glabrata*	400
	*C. parapsilosis*	400
	*C. tropicalis*	300

**Table 3:** Metabolites exhibiting a fold change greater than 2.

Heavy Metal	Compound Identified
Cu	(1S,3S)-6,8-dimethoxy-1,3-dimethyl-3,4-dihydro-1H-2-benzopyran-5-ol
	Tricyclo[3.3.3.0(1,5)]undec-6-ene-2,3,6-tricarbonitrile
	2-(β-D-Galactopyranosylthio)-6,7,8,9,10-pentahydrocycloheptathieno[2,3-d]-pyrimidine-4-thione
	2-(β-D-Glucopyranosylthio)-6,7,8,9,10-pentahydrocycloheptathieno[2,3-d]-pyrimidine-4-thione
	2-[5-(3-methoxyphenyl)-1,3,4-oxadiazol-2-yl]phenol
	9-Azatetracyclo[10.2.1.0(2,11).0(3,8)]pentadeca-3,5,7-triene-7-carboxylic acid
	10-(2,5-difluorophenyl) Benzene
	2-methoxy-4-(2-propenyl)-1-(1-propynyloxy)
Zn	4-(3-Pyridyl)thieno[2,3-d]pyridazin-7(6H)-one
	9-Azatetracyclo[10.2.1.0(2,11).0(3,8)]pentadeca-3,5,7-triene-7-carboxylic acid
	10-(2,3-difluorophenyl), E-15-Heptadecenal
	Hexacosane
	Hexadecane, 1-iodo
Cd	(2s,3r)-3-(1-naphthyl)glutamic acid
	2-propenoic acid, tridecyl ester
	5,7-dimethyl-1,2,3,4-tetrahydro-9-acridinamine
	Benzenamine, 2-bromo-4,6-dinitro
	Benzene
	Pentadecane

**Table 4:** Classification of metabolites identified in *Candida* metabolome after exposure to heavy metals, along with their composition. The quality score values for each metabolite are included in parenthesis.

Hydrocarbons
1-Docosene (81)
Hexadecane (83)
Octadecane, 1-iodo (80)
**Fatty acids**
Octadecanoic acid (94)
Octanoic acid (99)
Isopropyl myristate (95)
3-(4-N,N-Dimethylaminophenyl)propenoic acid, 2-(diethoxyphosphinyl)-, ethyl (91)
3,4-Dimethylbenzoic acid (93)
**Heterocyclic aromatic**
1H-Indole (93)
2-(1,3-Benzodioxol-5-ylmethyl)-1H-isoindole-1,3(2H)-dione (90)
Ethyl 2-(Chloromethyl)-2,3-dihydrobenzofuran-7-carboxylate (86)
**Substituted aromatics**
(3E,5E,7E,9E)-10-Phenyldeca-3,5,7,9-tetraen-2-ol (94)
4-Methoxy-N-(2-[(4-methoxyphenyl)imino]ethylidene)aniline (93)
**Non-aromatic cyclic compounds**
4-Isopropyl-1,3-cyclohexanedione (78)
**Organosiloxanes**
Tetracosamethylcyclododecasiloxane (90)

## Data Availability

The original contributions presented in this study are included in the article. Further inquiries can be directed to the corresponding author.
